# Hygenic and Miscellaneous

**Published:** 1871-03

**Authors:** 


### HYGENIC.

Dizziness—Its Cause and Prevention.—The condition existing in cases of dizziness is an unnatural pressure of the blood upon the brain. The exciting causes are usually some sudden exertion or hurried motion, as rising quickly from a recumbent position, stooping, etc. Unaccustomed motions, as sailing, swinging, walking circularly, riding backward, etc., often occasion it, even in healthy persons. The predisposing causes are either such as produce an undue thickness or viscidity of the blood, as torpidity of the excretory organs, particularly the liver, and excessive alimentation, or those that cause an unnatural thinness of the blood and weakness of circulation, as hemorrhage, an insufficient quantity of food, protracted disease, etc. The preventive treatment should be such as to restore the blood to its normal condition and equalize the circulation. Where the blood is thick and impure, the diet should be very plain and simple in quality—composed largely of fruit—and abstemious in quantity. Active out-door exercise and a thorough course of bathing are also indicated. Where the opposite condition exists, the indications are to nourish and strengthen the body, and equalize and invigorate the circulation. To this end the patient should have an abundance of pure air, as much nourishing food as can be digested and assimilated, and a moderate amount of daily exercise.—*Herald of Health.*

Exciting Causes of Diseases to be Avoided.—Persons who have any reason to suspect that they are subject to disease of the heart, should scrupulously avoid any exciting cause, either mental or physical, should not engage in any occupation entailing exciting exertions upon them, and should avoid the use of tobacco, and stimulants of all kinds, as a slight affection of the heart, which might otherwise cause but little trouble for a long series of years, will speedily be developed into active existence, by the frequent repetition of exciting causes.

Food for Nervous Dyspeptics.—Dyspeptics generally should adopt the two meal-a-day system, and eat nothing whatever at any other time. Let the breakfast be composed of oat-meal mush, quite dry, or oatmeal cakes and fruit, or unleavened graham bread or crackers and fruit. But one kind of fruit should be eaten at a meal, and that should be fresh and well ripened. For dinner, some of the articles mentioned for breakfast may be eaten, or some kind of vegetable that best agrees with the patient may be substituted for the fruit; or, if meat is eaten, lean beef or mutton and some vegetable. No other meats allowable, and as a rule, nervous dyspeptics are better off without any meat. No butter or greasy food of any kind, sugar, salt, spices, or condiments should be used. The patient MUST eat very slow and masticate his food very thoroughly. There is no rule more important than this. He should drink nothing whatever at meals, or for two hours afterward. He should not eat more than two kinds of food at a meal, and should never eat when in the least tired or excited. Cheerful company at meals is very important, and a hearty laugh, either at or after meals, is a great aid to digestion. "Laugh and grow fat" means to laugh and cease to be a dyspeptic.—*Herald of Health.*

Tooth Wash.—The mouth has a temperature of ninety-eight degrees, warmer than is ever experienced in the shade in the latitude of New England. It is well known that if beef, for example, be exposed in the shade during the warmest of our summer days. it will very soon begin to decompose. If we eat beef for dinner, the particles invariably find their way into the spaces between the teeth. Now, if these particles of beef are not removed, they will frequently remain till they are softened by decomposition. In most mouths this process of decomposition is in constant progress. Ought we to be surprised that the gums and teeth against which decomposing or putrifying masses lie should become subjects of disease? Much has been said *pro* and *con* upon the use of soap with the tooth-brush. My own experience, and the experience of members of my family, is highly favorable to the regular morning and evening use of soap.

Castile or other good soap will answer this purpose. (Whatever is good for the hands and face is good for the teeth.) The slight unpleasant taste which soap has when we begin to use it will be unnoticed. You have observed upon the teeth a yellow deposit, sometimes a black substance near the gums. If you examine either of them with a strong microsoope, you will find it all alive with animalculæ. These small animals live, keep house, and raise families of children, and die in your mouths. Nothing that can be safely introduced into the mouth checks them like soap.—*Medical Investigator.*

We would ask the writer of the above if he has examined the deposit on the teeth with a microscope for animalculæ? We do not doubt but living creatures are sometimes found in the tarter of the teeth, but it is far from being general. The importance of cleaning the teeth after each meal, however, is none the less, even if living creatures are never found in the tartar of the teeth.—*Herald of Health.*

Fish as an Article of Food.—Professor Agassiz, in his address before the Committee of the Legislature of Massachusetts, on the propagation and preservation of fishes, says, as reported in the *Boston Journal:* "The fish enters largely into the requisitions of the human system. It is a kind of food which refreshes the system, especially after intellectual fatigue. There is no other article of food that supplies the waste of the head so thoroughly as fish diet; and the evidence of it is in the fact that all the inhabitants of the sea-shores the world over are the brighter population of the country. Fish contains phosphorus to a large extent, a chemical element which the brain requires for growth and health. He would not say that an exclusive use of fish would make a blockhead a wise man, but that the brain should not be wanting in one of its essential elements."—(*St. Louis Medical Reporter.*)—*Dental Cosmos, August* 1869 *p.* 445.

The Virtue of the Sunflower.—Mr. Martin, in a paper presented by him to the *Societe Therapeutique France*, affirms that the common sunflower, extensively cultivated, has the effect of neutralizing the unwholesome vapors which are so fatal to health and life in marshy districts. The Dutch, who live only by dyking and draining their low lands, and are therefore good authority, pronounce sunflower culture a specific for intermittent fever, the scourge of Holland. They assert that it has disappeared from every district where the experiment has been tried. It is not yet known whether this is the result of its rapid growth producing oxygen, or whether it emits ozone and destroys those germs, animal and vegetable, which produce that miasma which brings fever in its train.—*Medical Record.*

VENTILATION.—It is more difficult to ventilate a close room in summer than in winter; because in summer there are no fires to create a draft, or to move the air; but an open fireplace, or an open door, or long windows, open at top and bottom, may be sufficient.

HOW TO TRAVEL FOR HEALTH.—The best mode of travel is on foot or on a saddle; the next best, is in an open carriage, or in an old-fashioned stage coach. A sea voyage, and almost any mode of travelling by water, is, in general useful; but it would be a serious practical joke if any one were to advise an invalid to seek health in a railroad car.

INSANITY AND POOR DIET.—In England and Wales, out of 54,713 persons of unsound mind, 48,325 were of the pauper class, and the Lunacy Commissioners report that in a great majority of cases impaired nutrition was the cause of the malady. Upon bodies and minds thus reduced, griefs and perplexities act with most damaging influence. Wholesome food, and a good stomach to elaborate it, would keep a majority of the race sane in mind and body.—*Herald of Health.*

THE USE OF RAW MEAT IN DIARRHŒA AND DYSPEPSIA—Dr. Robert Druitt, M. R. C. P. (*Medical Times and Gazette*), first learned the use of raw meat as a remedy for diarrhœa, from the late Professor Trousseau, in 1851, and since that visit to Paris he has had, with others, abundant opportunities of proving its efficacy. It is useful in infantile "cholera," chronic diarrhœas of children, habitual diarrhœa associated with "marasmus," obstinate vomiting of pregnancy, cases of atrophy, dyspepsia, and mal-nutrition.

WHEN TO GIVE ARTIFICIAL FOOD TO INFANTS.—Dr. Brinton (*Trans. Lond. Obstet. Soc.*), advocates giving artificial food to babies, such as corn-flour with milk, when the first teeth come and the child begins to bite its mother's nipple. When the child's mouth is toothless, its office is to suck the breasts. He orders milk and water until the teeth appear, to infants brought up by hand.

LIVER AS FOOD.—The liver and kidneys are the most unhealthful parts of an animal, for the reason that they are constantly charged with the worn out excrementitious matters of the system. This is why they are the parts first subject to decomposition.—*Herald of Health.*

Position of the Head During Sleep.—"Should the head be elevated or upon a level with the body during sleep; or, in other words, are pillows and bolsters useful or injurious?"

The head should be in the same relative position with the body during sleep as when the person is sitting or standing. Consequently, when lying upon the back, no pillow or bolster is needed. When lying upon the side, a pillow of sufficient size to keep the head upon a line with the spine should be used. If the head is raised above the level of the body, both respiration and the circulation of the blood are interfered with in proportion to the degree of elevation. The air passes to and from the lungs, and the blood to and from the heart, in tubes contained in the neck. Now take a straight tube of any kind, and a certain quantity of air or liquid, at a certain pressure, will pass through it in a given time; but if you bend the tube as the tubes in the neck are bent by having the head elevated, the quantity of air or liquid which will pass through in the same time with the same pressure will be diminished, and the greater the bend the greater the obstruction. Twisting of the neck also interferes with the respiration and circulation by diminishing the capacity of the trachea and blood-vessels.—*Herald of Health.*

Duration of Pregnancy.—M. Aubinais (Medical Gazette, Sept. 1870, p. 176; from Journ. de Med. et de Chirurg,) cites the case of two sisters, of irreproachable virtue, who on the same day were married to two sailors. At six o'clock, the next morning the husbands were ordered away to their ship. On the two hundred and sixty-fourth day after their marriage the two women were brought to bed at almost the same hour and under the same roof. The possibility of fecundation of a virgin by a single act of coition cannot, therefore, be denied, although it is an exceptional fact.

Hysterical Retention of Urine.—Mr. J. Waring-Curran (Medical Press and Circular,) finds that this troublesome affection can be relieved by causing the sufferers suddenly to plunge their hands and arms into very cold water.

Syphilitic Infction from Kissing.—Dr. M. F. Maury says (Med. and Surg. Reporter,): "I have seen two cases, one now on hand in my private practice, where a patient with mucous patches upon the lip, kissing another, developed upon the lip of the latter a hard chancre, with its attendants of indurated base, involvement of the submaxillary lymphatic ganglions, of the affected side, and a profuse secondary manifestation of roseola maculata."

THE PULSE.—The pulse was first noticed by Galen. It is slower in the inhabitant of the country than in one of the city. In the West Indies it is 100 ; in Greenland, as slow as 40 ; and is slowest in the cold climates. It is slower in the winter than in summer. Is slow in the morning, quick at noon, and more frequent at night.—*Med. and Surg. Reporter.*

*Cure for Corns.*—Soak the feet well in warm water, then with a sharp instrument pare off as much of the corn as can be done without pain, and bind up the part affected with a piece of linen or muslin thoroughly saturated with sperm oil, or what is better, the oil which floats upon the surface of the pickle of herring or mackerel. After three or four days the dressing may be removed, and the remaining dead cuticle removed by scraping, when the new skin will be found of a soft and healthy texture and less liable to the formation of a new corn than before. We have this recipe from a source that we cannot well doubt, and publish it for the benefit of many suffering readers.—*Journal of Applied Chemistry.*—*Nashville Journal of Medicine and Surgery*, Jan. 1870.

*Hereditary Twin-bearing Family.*—Dr. Curgenven (Trans. Obstetrical Soc. of London) attended a lady who was delivered of twins for the fourth time ; she had severe flooding, and barely escaped with her life. This was her sixteenth conception during a married life of sixteen years. Her mother had twins once, her aunt once, and her great-grandmother twice.

*The Originator of Pathological Anatomy.*—About three centuries before the Christian era, Herophilus, a native of Chalcedon, and Erasistratus, of Inlis, a grandson of Aristotle, laid the foundation-stones of the science of human anatomy. Herophilus, it has been said, resorted to human vivisections, more particularly of criminals. He was the originator of pathological anatomy, and was the first to propose post-mortem examination to learn the cause of death. Fallopius, centuries after, denominated him "the evangelist of anatomists."—*Dr. Gouvr. M. Smith's Discourse.*

*Remedy for Toothache.*—℞—Spirit ammonia, ℥ ss., chloroform ℥ i, oil caryophyl ℥ ii, Tinc. camphor, ℥ ii. m. After cleaning out and drying the cavity, a small pellet of raw cotton should be moistened with this, and applied to the exposed pulp, repeating the application as occasion requires.

HYPOPHOSPHITE OF SODA.—Dr. Jno. C. Thorowgood thinks this salt answers all the purposes of pure phosphorous as an internal remedy, and as a gradual tonic and restorer of nerve force. In cases of nervous depression and torpor, with, at times, shooting neuralgic pains; or in other cases, numbness and deadness of the limbs, as from feeble circulation, the hypophosphites prove useful, and the lime or soda salt can be given according to the way in which the stomach may seem to bear the one better than the other. When anæmia is present, the citrate of iron can be added, or else the syrup of the hypophosphites of iron, or iron with quinine, can be employed. Either of these syrups will prove an active tonic, removing neuralgic pains, chest oppression, and languor of circulation in a very evident way.—

*Carbolic Acid.*—Applied to hemorrhoids, carbolic acid corrugates the sac. It will destroy pediculi of all kinds. The full strength is escharotic, but diluted in glycerin (5 drops to the ounce) it forms an agreeable application.

*Removing particles of foreign matter from the Eye.*—The late Dr. Munn's process was, to scarify the lid in the usual way, then close the eye, and allow the blood to coagulate and entangle the irritating substance in the clot. When the latter is removed, it brings away the foreign particles with it.—*Medical and Surgical Reporter.*

*Cure for Warts.*—Warts may be readily cured by cutting a hole in a piece of sticking plaster, so that the wart protrudes, and the using—

| | | |
|---|---|---|
| ℞—Caustic Potash, | . . . . | ℥ ij. |
| Gum Arabic, | . . . . | ℥ ss. |
| Flour, | . . . . | q. s. m. |

Make a paste. S. To be left in a few hours.—*Boston Journal of Chemistry.*

*Chromic Acid in Ringworm*—One or two applications of a solution of chromic acid (one drachm to the ounce of water,) has proved very efficacious in curing the disease.—*Am. Practitioner.*

*Cure for Obesity.*—Dr. C. D. Gib says obesity may be cured by taking, for a considerable time, very small doses of bromide of ammonium.

*Incontinence of Urine.*—Dr. W. Thompson, (Lancet, November 1870, p. 703,) highly recommends a dose of chloral at bedtime for this disease in children.

*Migraine—One side headache.*—Muriate of ammonia, in doses of from ten to twenty grains, will be found an excellent remedy.

